# Poly[[aqua­(μ_2_-oxalato)(μ_2_-2-oxido­pyridinium-3-carboxylato)holmium(III)] monohydrate]

**DOI:** 10.1107/S1600536809036344

**Published:** 2009-09-16

**Authors:** Hui-Lan Zhu, Hui-Ling Lai, Lu Han, Yi-Fan Luo, Rong-Hua Zeng

**Affiliations:** a School of Chemistry and Environment, South China Normal University, Guangzhou 510006, People’s Republic of China; b Key Laboratory of Technology on Electrochemical Energy Storage and Power Generation in Guangdong Universities, South China Normal University, Guangzhou 510006, People’s Republic of China

## Abstract

In the title complex, {[Ho(C_2_O_4_)(C_6_H_4_NO_3_)(H_2_O)]·(H_2_O)}_*n*_, the Ho^III^ ion is coordinated by three O atoms from two 2-oxidopyridinium-3-carboxylate ligands, four O atoms from two oxalate ligands and one water mol­ecule in a distorted bicapped trigonal-prismatic geometry. The 2-oxidopyridin­ium-3-carboxylate and oxalate ligands link the Ho^III^ ions into a layer in (100). These layers are further connected by inter­molecular O—H⋯O hydrogen bonds involving the coordinated water mol­ecules to assemble a three-dimensional supra­molecular network. The uncoordin­ated water mol­ecule is involved in N—H⋯O and O—H⋯O hydrogen bonds within the layer.

## Related literature

For general background to mol­ecular self-assembly of supra­molecular architectures, see: Deng *et al.* (2008 ▶); Zeng *et al.* (2007 ▶). For 2-oxidopyridinium-3-carboxylic acid and oxalic acid as building blocks, see: Huang *et al.* (2009 ▶).
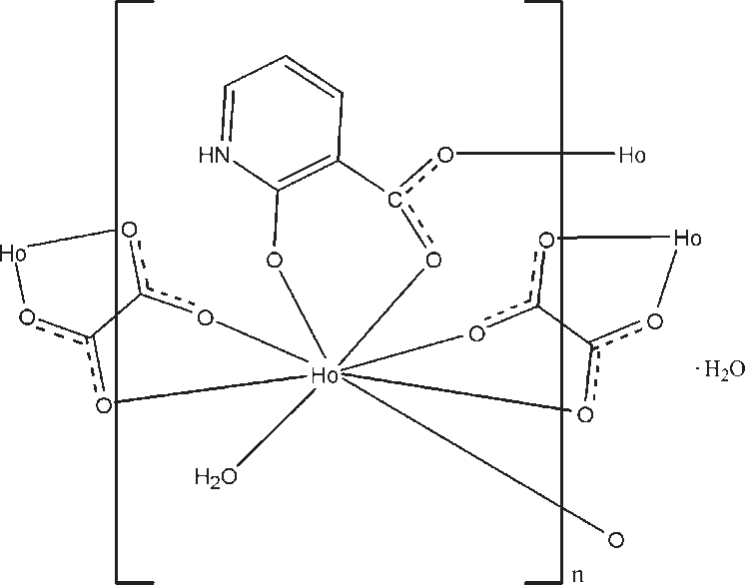


## Experimental

### 

#### Crystal data

[Ho(C_2_O_4_)(C_6_H_4_NO_3_)(H_2_O)]·H_2_O
*M* *_r_* = 427.08
Triclinic, 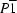

*a* = 6.5391 (11) Å
*b* = 9.5305 (16) Å
*c* = 9.7391 (16) Å
α = 71.810 (2)°
β = 78.862 (2)°
γ = 80.359 (2)°
*V* = 562.01 (16) Å^3^
*Z* = 2
Mo *K*α radiation
μ = 7.09 mm^−1^
*T* = 296 K
0.20 × 0.18 × 0.17 mm

#### Data collection

Bruker APEXII CCD diffractometer
Absorption correction: multi-scan (*SADABS*; Sheldrick, 1996 ▶) *T*_min_ = 0.332, *T*_max_ = 0.379
2884 measured reflections
1983 independent reflections
1862 reflections with *I* > 2σ(*I*)
*R*_int_ = 0.018

#### Refinement

*R*[*F* ^2^ > 2σ(*F* ^2^)] = 0.034
*wR*(*F* ^2^) = 0.090
*S* = 1.06
1983 reflections
184 parameters
6 restraints
H atoms treated by a mixture of independent and constrained refinement
Δρ_max_ = 2.75 e Å^−3^
Δρ_min_ = −2.19 e Å^−3^

### 

Data collection: *APEX2* (Bruker, 2007 ▶); cell refinement: *SAINT* (Bruker, 2007 ▶); data reduction: *SAINT*; program(s) used to solve structure: *SHELXS97* (Sheldrick, 2008 ▶); program(s) used to refine structure: *SHELXL97* (Sheldrick, 2008 ▶); molecular graphics: *DIAMOND* (Brandenburg, 1999 ▶); software used to prepare material for publication: *SHELXL97*.

## Supplementary Material

Crystal structure: contains datablocks I, global. DOI: 10.1107/S1600536809036344/hy2226sup1.cif

Structure factors: contains datablocks I. DOI: 10.1107/S1600536809036344/hy2226Isup2.hkl

Additional supplementary materials:  crystallographic information; 3D view; checkCIF report

## Figures and Tables

**Table 1:** Selected bond lengths (Å)

Ho1—O1	2.354 (5)
Ho1—O2^i^	2.348 (5)
Ho1—O3	2.279 (5)
Ho1—O4	2.406 (4)
Ho1—O5^ii^	2.364 (5)
Ho1—O6	2.394 (5)
Ho1—O7^iii^	2.392 (5)
Ho1—O1*W*	2.348 (5)

Symmetry codes: (i) 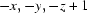; (ii) 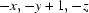; (iii) 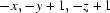.

**Table 2:** Hydrogen-bond geometry (Å, °)

*D*—H⋯*A*	*D*—H	H⋯*A*	*D*⋯*A*	*D*—H⋯*A*
O1*W*—H1*W*⋯O2^iv^	0.84 (6)	2.02 (6)	2.737 (7)	142 (8)
O1*W*—H2*W*⋯O4^v^	0.85 (7)	1.96 (4)	2.749 (7)	154 (8)
O2*W*—H3*W*⋯O6	0.84 (7)	2.36 (11)	2.882 (10)	121 (8)
O2*W*—H4*W*⋯O1	0.86 (7)	2.27 (3)	3.082 (8)	157 (9)
N1—H1⋯O2*W*^vi^	0.86	1.99	2.781 (10)	154

Symmetry codes: (iv) 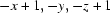; (v) 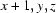; (vi) 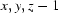.

**Fig. 1.**
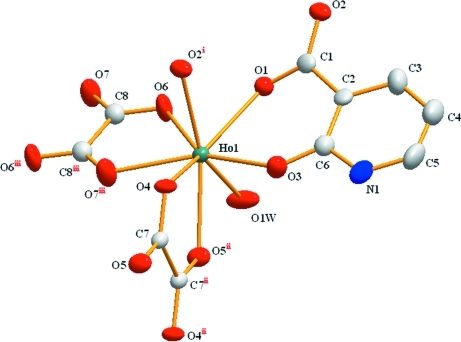
The asymmetric unit of the title compound. Displacement ellipsoids are drawn at the 30% probability level. H atoms have been omitted for clarity. [Symmetry codes: (i) -x, -y, 1 - z; (ii) -x, 1 - y, -z; (iii) -x, 1 - y, 1 - z.]

**Fig. 2.**
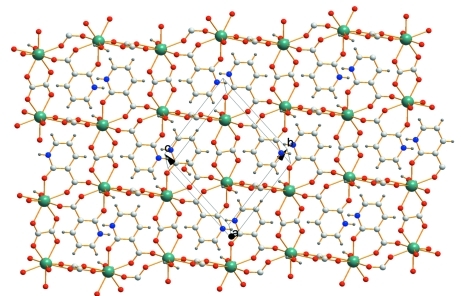
View of a layered network of the title compound.

## supplementary crystallographic information

### Comment

Molecular self-assembly of supramolecular architectures has received much attention during recent decades (Deng *et al.*, 2008; Zeng *et al.*, 2007). The structures and properties of such systems depend on the coordination and geometric preferences of both the central metal ions and the bridging building blocks, as well as the influence of weaker non-covalent interactions, such as hydrogen bonds and π–π stacking interactions. As a building block, 2-oxynicotinic acid and oxalic acid are good ligands with multifunctional coordination sites providing a high likelihood for the generation of structures with high dimensions (Huang *et al.*, 2009). Recently, we obtained the title coordination polymer, which was synthesized under hydrothermal conditions.

In the title compound (Fig. 1), the Ho^III^ centre is eight-coordinated by seven O atoms from two 2-oxynicotinate ligands and two oxalate ligands, and by one water molecule in a distorted bicapped trigonal prismatic geometry, with Ho—O distances and O—Ho—O angles ranging from 2.279 (5) to 2.406 (4) Å and 67.30 (17) to 148.26 (17)°, respectively (Table 1). The carboxylate groups of the 2-oxynicotinates and oxalates act as bridging ligands, linking the Ho centres into a layer parallel to the (100) plane (Fig. 2). The layers are further connected by intermolecular O—H···O hydrogen bonds involving the coordinated water molecules into a three-dimensional supramolecular network (Table 2). The uncoordinated water molecule is involved in N—H···O and O—H···O hydrogen bonds within the layer (Table 2).

### Experimental

A mixture of Ho_2_O_3_ (0.375 g, 1 mmol), 2-oxynicotinic acid (0.127 g, 1 mmol), oxalic acid (0.09 g, 1 mmol), water (10 ml) in the presence of HNO_3_ (0.024 g, 0.385 mmol) was stirred vigorously for 20 min and then sealed in a Teflon-lined stainless-steel autoclave (20 ml capacity). The autoclave was heated and maintained at 446 K for 2 d, and then cooled to room temperature at 5 K h^-1^, giving colourless block crystals.

### Refinement

Water H atoms were tentatively located in difference Fourier maps and refined with distance restraints of O—H = 0.84 (1) and H···H = 1.35 (1) Å, and with a fixed *U*_iso_(H) = 0.064 Å^2^. H atoms attached to C and N atoms were placed at calculated positions and treated as riding on their parent atoms, with C—H = 0.93 and N—H= 0.86 Å and with *U*_iso_(H) = 1.2*U*_eq_(C,N). The highest residual electron density was found 0.94 Å from Ho1 and the deepest hole 0.91 Å from Ho1.

### Crystal data

**Table:**

[Ho(C_2_O_4_)(C_6_H_4_NO_3_)(H_2_O)]·H_2_O	*Z* = 2
*M**_r_* = 427.08	*F*(000) = 404
Triclinic, *P*1	*D*_x_ = 2.524 Mg m^−^^3^
Hall symbol: -P 1	Mo *K*α radiation, λ = 0.71073 Å
*a* = 6.5391 (11) Å	Cell parameters from 2285 reflections
*b* = 9.5305 (16) Å	θ = 2.2–28.1°
*c* = 9.7391 (16) Å	µ = 7.09 mm^−^^1^
α = 71.810 (2)°	*T* = 296 K
β = 78.862 (2)°	Block, colourless
γ = 80.359 (2)°	0.20 × 0.18 × 0.17 mm
*V* = 562.01 (16) Å^3^	

### Data collection

**Table:**

Bruker APEXII CCD diffractometer	1983 independent reflections
Radiation source: fine-focus sealed tube	1862 reflections with *I* > 2σ(*I*)
graphite	*R*_int_ = 0.018
φ and ω scans	θ_max_ = 25.2°, θ_min_ = 2.2°
Absorption correction: multi-scan (*SADABS*; Sheldrick, 1996)	*h* = −7→6
*T*_min_ = 0.332, *T*_max_ = 0.379	*k* = −11→11
2884 measured reflections	*l* = −11→10

### Refinement

**Table:**

Refinement on *F*^2^	Primary atom site location: structure-invariant direct methods
Least-squares matrix: full	Secondary atom site location: difference Fourier map
*R*[*F*^2^ > 2σ(*F*^2^)] = 0.034	Hydrogen site location: inferred from neighbouring sites
*wR*(*F*^2^) = 0.090	H atoms treated by a mixture of independent and constrained refinement
*S* = 1.06	*w* = 1/[σ^2^(*F*_o_^2^) + (0.0695*P*)^2^] where *P* = (*F*_o_^2^ + 2*F*_c_^2^)/3
1983 reflections	(Δ/σ)_max_ < 0.001
184 parameters	Δρ_max_ = 2.75 e Å^−^^3^
6 restraints	Δρ_min_ = −2.19 e Å^−^^3^

### Fractional atomic coordinates and isotropic or equivalent isotropic displacement parameters (Å^2^)

**Table:**

	*x*	*y*	*z*	*U*_iso_*/*U*_eq_	
Ho1	0.09083 (4)	0.29059 (3)	0.30045 (3)	0.01784 (15)	
O4	−0.1655 (7)	0.3863 (5)	0.1370 (5)	0.0219 (10)	
O3	0.1549 (8)	0.1295 (6)	0.1626 (5)	0.0311 (11)	
O6	0.1351 (9)	0.3228 (5)	0.5267 (6)	0.0338 (12)	
O1	0.2127 (8)	0.0581 (5)	0.4520 (5)	0.0251 (10)	
O5	−0.2292 (7)	0.5458 (5)	−0.0767 (5)	0.0258 (10)	
N1	0.2729 (11)	−0.0557 (8)	0.0581 (7)	0.0366 (15)	
H1	0.2407	0.0054	−0.0227	0.044*	
C6	0.2307 (12)	−0.0067 (8)	0.1802 (8)	0.0266 (15)	
C8	0.0606 (11)	0.4422 (8)	0.5578 (8)	0.0261 (15)	
C5	0.3624 (14)	−0.1948 (10)	0.0574 (10)	0.044 (2)	
H5	0.3862	−0.2213	−0.0290	0.052*	
C4	0.4170 (14)	−0.2947 (9)	0.1807 (10)	0.048 (3)	
H4	0.4851	−0.3882	0.1795	0.057*	
C3	0.3695 (13)	−0.2550 (9)	0.3094 (10)	0.0396 (19)	
H3	0.3999	−0.3250	0.3960	0.048*	
C2	0.2776 (10)	−0.1133 (7)	0.3126 (7)	0.0251 (15)	
C1	0.2306 (10)	−0.0747 (7)	0.4529 (7)	0.0236 (14)	
C7	−0.1147 (10)	0.4807 (7)	0.0161 (7)	0.0190 (13)	
O2W	0.2278 (13)	0.0635 (9)	0.7646 (8)	0.0621 (19)	
O2	0.2125 (7)	−0.1813 (5)	0.5703 (5)	0.0262 (10)	
O7	0.0730 (9)	0.4748 (6)	0.6691 (5)	0.0358 (13)	
O1W	0.4526 (8)	0.2970 (7)	0.2822 (6)	0.0427 (15)	
H3W	0.164 (16)	0.149 (7)	0.758 (10)	0.064*	
H4W	0.203 (16)	0.041 (10)	0.691 (6)	0.064*	
H1W	0.508 (12)	0.263 (11)	0.359 (5)	0.064*	
H2W	0.549 (10)	0.332 (11)	0.215 (6)	0.064*	

### Atomic displacement parameters (Å^2^)

**Table:**

	*U*^11^	*U*^22^	*U*^33^	*U*^12^	*U*^13^	*U*^23^
Ho1	0.0166 (2)	0.0197 (2)	0.0158 (2)	−0.00221 (12)	−0.00168 (12)	−0.00363 (13)
O4	0.018 (2)	0.027 (2)	0.017 (2)	−0.0088 (18)	−0.0019 (18)	0.0028 (19)
O3	0.039 (3)	0.031 (3)	0.020 (2)	0.002 (2)	−0.005 (2)	−0.007 (2)
O6	0.046 (3)	0.023 (3)	0.034 (3)	0.010 (2)	−0.013 (2)	−0.013 (2)
O1	0.027 (3)	0.024 (2)	0.022 (2)	−0.0029 (19)	−0.0024 (19)	−0.0050 (19)
O5	0.016 (2)	0.035 (3)	0.023 (2)	−0.0035 (19)	−0.0071 (19)	−0.001 (2)
N1	0.040 (4)	0.043 (4)	0.028 (3)	−0.009 (3)	0.003 (3)	−0.016 (3)
C6	0.025 (4)	0.027 (4)	0.029 (4)	−0.007 (3)	−0.001 (3)	−0.010 (3)
C8	0.024 (4)	0.026 (4)	0.028 (4)	−0.005 (3)	−0.003 (3)	−0.007 (3)
C5	0.048 (5)	0.046 (5)	0.042 (5)	−0.011 (4)	0.014 (4)	−0.028 (4)
C4	0.055 (6)	0.036 (5)	0.050 (6)	−0.008 (4)	0.013 (5)	−0.021 (5)
C3	0.038 (5)	0.032 (4)	0.047 (5)	0.000 (3)	0.001 (4)	−0.015 (4)
C2	0.018 (3)	0.025 (3)	0.029 (4)	−0.008 (3)	0.007 (3)	−0.006 (3)
C1	0.012 (3)	0.026 (3)	0.028 (4)	0.000 (3)	−0.003 (3)	−0.003 (3)
C7	0.018 (3)	0.020 (3)	0.020 (3)	−0.006 (2)	0.001 (3)	−0.007 (3)
O2W	0.069 (5)	0.078 (5)	0.041 (4)	−0.022 (4)	−0.020 (4)	−0.005 (4)
O2	0.019 (2)	0.027 (3)	0.027 (3)	−0.0043 (19)	−0.0037 (19)	0.002 (2)
O7	0.056 (4)	0.031 (3)	0.024 (3)	0.008 (2)	−0.018 (2)	−0.013 (2)
O1W	0.021 (3)	0.062 (4)	0.029 (3)	−0.012 (3)	−0.008 (2)	0.015 (3)

### Geometric parameters (Å, °)

**Table:**

Ho1—O1	2.354 (5)	C6—C2	1.421 (10)
Ho1—O2^i^	2.348 (5)	C8—O7	1.237 (9)
Ho1—O3	2.279 (5)	C8—C8^iii^	1.546 (15)
Ho1—O4	2.406 (4)	C5—C4	1.347 (13)
Ho1—O5^ii^	2.364 (5)	C5—H5	0.9300
Ho1—O6	2.394 (5)	C4—C3	1.384 (11)
Ho1—O7^iii^	2.392 (5)	C4—H4	0.9300
Ho1—O1W	2.348 (5)	C3—C2	1.390 (10)
O4—C7	1.262 (8)	C3—H3	0.9300
O3—C6	1.279 (9)	C2—C1	1.487 (10)
O6—C8	1.263 (9)	C1—O2	1.271 (8)
O1—C1	1.249 (8)	C7—C7^ii^	1.554 (12)
O5—C7	1.232 (8)	O2W—H3W	0.84 (7)
O5—Ho1^ii^	2.364 (4)	O2W—H4W	0.86 (7)
N1—C5	1.358 (11)	O1W—H1W	0.84 (6)
N1—C6	1.373 (10)	O1W—H2W	0.85 (7)
N1—H1	0.8600		
			
O3—Ho1—O1W	90.4 (2)	C5—N1—C6	123.9 (7)
O3—Ho1—O2^i^	90.37 (18)	C5—N1—H1	118.0
O1W—Ho1—O2^i^	148.26 (17)	C6—N1—H1	118.0
O3—Ho1—O1	73.45 (17)	O3—C6—N1	116.8 (7)
O1W—Ho1—O1	75.15 (18)	O3—C6—C2	127.2 (7)
O2^i^—Ho1—O1	74.69 (16)	N1—C6—C2	116.1 (7)
O3—Ho1—O5^ii^	81.80 (17)	O7—C8—O6	127.4 (7)
O1W—Ho1—O5^ii^	67.83 (16)	O7—C8—C8^iii^	117.7 (8)
O2^i^—Ho1—O5^ii^	143.49 (16)	O6—C8—C8^iii^	114.9 (8)
O1—Ho1—O5^ii^	134.91 (16)	C4—C5—N1	120.6 (8)
O3—Ho1—O7^iii^	148.06 (18)	C4—C5—H5	119.7
O1W—Ho1—O7^iii^	105.7 (2)	N1—C5—H5	119.7
O2^i^—Ho1—O7^iii^	89.77 (18)	C5—C4—C3	118.5 (8)
O1—Ho1—O7^iii^	136.83 (16)	C5—C4—H4	120.7
O5^ii^—Ho1—O7^iii^	79.25 (18)	C3—C4—H4	120.7
O3—Ho1—O6	144.55 (17)	C4—C3—C2	121.7 (8)
O1W—Ho1—O6	75.0 (2)	C4—C3—H3	119.1
O2^i^—Ho1—O6	86.35 (18)	C2—C3—H3	119.1
O1—Ho1—O6	71.64 (16)	C3—C2—C6	119.1 (7)
O5^ii^—Ho1—O6	119.81 (18)	C3—C2—C1	120.1 (7)
O7^iii^—Ho1—O6	67.30 (17)	C6—C2—C1	120.8 (6)
O3—Ho1—O4	77.17 (17)	O1—C1—O2	122.8 (6)
O1W—Ho1—O4	135.57 (16)	O1—C1—C2	119.9 (6)
O2^i^—Ho1—O4	75.25 (15)	O2—C1—C2	117.3 (6)
O1—Ho1—O4	137.32 (16)	O5—C7—O4	126.5 (6)
O5^ii^—Ho1—O4	68.24 (14)	O5—C7—C7^ii^	117.4 (7)
O7^iii^—Ho1—O4	72.00 (17)	O4—C7—C7^ii^	116.0 (7)
O6—Ho1—O4	135.14 (16)	H3W—O2W—H4W	105 (9)
C7—O4—Ho1	118.2 (4)	C1—O2—Ho1^i^	128.4 (4)
C6—O3—Ho1	136.0 (5)	C8—O7—Ho1^iii^	119.8 (5)
C8—O6—Ho1	120.3 (5)	Ho1—O1W—H1W	119 (6)
C1—O1—Ho1	136.8 (4)	Ho1—O1W—H2W	136 (6)
C7—O5—Ho1^ii^	119.9 (4)	H1W—O1W—H2W	106 (3)

Symmetry codes: (i) −*x*, −*y*, −*z*+1; (ii) −*x*, −*y*+1, −*z*; (iii) −*x*, −*y*+1, −*z*+1.

### Hydrogen-bond geometry (Å, °)

**Table:**

*D*—H···*A*	*D*—H	H···*A*	*D*···*A*	*D*—H···*A*
O1W—H1W···O2^iv^	0.84 (6)	2.02 (6)	2.737 (7)	142 (8)
O1W—H2W···O4^v^	0.85 (7)	1.96 (4)	2.749 (7)	154 (8)
O2W—H3W···O6	0.84 (7)	2.36 (11)	2.882 (10)	121 (8)
O2W—H4W···O1	0.86 (7)	2.27 (3)	3.082 (8)	157 (9)
N1—H1···O2W^vi^	0.86	1.99	2.781 (10)	154

Symmetry codes: (iv) −*x*+1, −*y*, −*z*+1; (v) *x*+1, *y*, *z*; (vi) *x*, *y*, *z*−1.

## References

[bb1] Brandenburg, K. (1999). *DIAMOND* Crystal Impact GbR, Bonn, Germany.

[bb2] Bruker (2007). *APEX2* and *SAINT* Bruker AXS Inc., Madison, Wisconsin, USA.

[bb3] Deng, H., Qiu, Y.-C., Li, Y.-H., Liu, Z.-H., Zeng, R.-H., Zeller, M. & Batten, S. R. (2008). *Chem. Commun.* pp. 2239–2241. 10.1039/b800938d 18463752

[bb4] Huang, C.-D., Huang, J.-X., Wu, Y.-Y., Lian, Y.-Y. & Zeng, R.-H. (2009). *Acta Cryst.* E**65**, m177–m178. 10.1107/S1600536809000580 PMC2968133 21581782

[bb5] Sheldrick, G. M. (1996). *SADABS* University of Göttingen, Germany.

[bb6] Sheldrick, G. M. (2008). *Acta Cryst.* A**64**, 112–122. 10.1107/S0108767307043930 18156677

[bb7] Zeng, R.-H., Qiu, Y.-C., Cai, Y.-P., Wu, J.-Z. & Deng, H. (2007). *Acta Cryst.* E**63**, m1666.

